# Comparative genomic and phenotypic characterization of invasive non-typhoidal *Salmonella* isolates from Siaya, Kenya

**DOI:** 10.1371/journal.pntd.0008991

**Published:** 2021-02-01

**Authors:** Jessica Z. Kubicek-Sutherland, Gary Xie, Migun Shakya, Priya K. Dighe, Lindsey L. Jacobs, Hajnalka Daligault, Karen Davenport, Loreen R. Stromberg, Zachary R. Stromberg, Qiuying Cheng, Prakasha Kempaiah, John Michael Ong’echa, Vincent Otieno, Evans Raballah, Samuel Anyona, Collins Ouma, Patrick S. G. Chain, Douglas J. Perkins, Harshini Mukundan, Benjamin H. McMahon, Norman A. Doggett

**Affiliations:** 1 Los Alamos National Laboratory, Los Alamos, New Mexico, United States; 2 Center for Global Health, University of New Mexico, Albuquerque, New Mexico, United States of America; 3 University of New Mexico/KEMRI Laboratories of Parasitic and Viral Diseases, Kenya Medical Research Institute, Kisumu, Kenya; 4 Department of Medical Laboratory Science, School of Public Health, Biomedical Sciences and Technology, Masinde Muliro University of Science and Technology, Kakamega, Kenya; 5 Department of Medical Biochemistry, School of Medicine, Maseno University, Maseno, Kenya; 6 Department of Biomedical Sciences and Technology, School of Public Health and Community Development, Maseno University, Maseno, Kenya; Yale University Yale School of Public Health, UNITED STATES

## Abstract

Non-typhoidal *Salmonella* (NTS) is a major global health concern that often causes bloodstream infections in areas of the world affected by malnutrition and comorbidities such as HIV and malaria. Developing a strategy to control the emergence and spread of highly invasive and antimicrobial resistant NTS isolates requires a comprehensive analysis of epidemiological factors and molecular pathogenesis. Here, we characterize 11 NTS isolates that caused bloodstream infections in pediatric patients in Siaya, Kenya from 2003–2010. Nine isolates were identified as *S*. Typhimurium sequence type 313 while the other two were *S*. Enteritidis. Comprehensive genotypic and phenotypic analyses were performed to compare these isolates to those previously identified in sub-Saharan Africa. We identified a *S*. Typhimurium isolate referred to as UGA14 that displayed novel plasmid, pseudogene and resistance features as compared to other isolates reported from Africa. Notably, UGA14 is able to ferment both lactose and sucrose due to the acquisition of insertion elements on the pKST313 plasmid. These findings show for the first time the co-evolution of plasmid-mediated lactose and sucrose metabolism along with cephalosporin resistance in NTS further elucidating the evolutionary mechanisms of invasive NTS phenotypes. These results further support the use of combined genomic and phenotypic approaches to detect and characterize atypical NTS isolates in order to advance biosurveillance efforts that inform countermeasures aimed at controlling invasive and antimicrobial resistant NTS.

## Author summary

Non-typhoidal *Salmonella* (NTS) has been associated with life-threatening bacteremia in sub-Saharan Africa where co-morbidities such as HIV and malaria are highly prevalent. Children under the age of 5 are especially vulnerable to invasive NTS infections. The emergence and spread of multi-drug resistant invasive NTS isolates have limited the availability of effective treatment options. Understanding the molecular mechanisms that drive the evolution of invasive and antimicrobial resistant NTS strains is key to mitigating their impact on human health. In this study, we obtained 11 NTS isolates from the bloodstreams of children in Siaya, Kenya and performed both genotypic and phenotypic characterization compared to antimicrobial sensitive NTS strains. One strain, named UGA14, displayed a unique plasmid makeup compared to the other 10 isolates, which encoded for cephalosporin resistance as well as novel metabolic features allowing it to metabolize both lactose and sucrose. Not only was UGA14 multi-drug resistant but its unique metabolic profile made it indistinguishable from *Escherichia coli* on brilliant green agar indicating the failure of traditional culture-based techniques to inform diagnosis and treatment decisions. These findings highlight the importance of comparative genotypic and phenotypic analyses to understand the driving mechanisms of invasive and drug-resistant pathogens and support the development of effective countermeasures.

## Introduction

Non-typhoidal *Salmonella* (NTS) infections are of global concern, causing symptoms ranging from limited gastroenteritis to life-threatening bacteremia [1]. Invasive NTS infections are associated with a mortality rate of 20–30% in children below 5 years of age in sub-Saharan Africa, including rural Kenya, which is the epicenter of the study presented here [2,3]. While there are over 2500 *Salmonella enterica* serovars, Typhimurium and Enteritidis are the two that are frequently transmitted from animals to humans in most parts of the world [4]. NTS causes a high burden to the developing world due to the severity of illness (especially in young children) and the ease of transmissibility [5]. The severity of illness is further exacerbated by the high incidence of extensive antimicrobial resistance (AMR) in NTS [6,7], especially in sub-Saharan African countries such as Kenya [8,9]. NTS is more prevalent in immunocompromised individuals, and risk factors include extremes of age, alteration of the endogenous microbiota of the intestine (e.g. as a result of antimicrobial therapy or surgery), diabetes, malignancy, rheumatological disorders, reticuloendothelial blockade (e.g. as a result of malaria and sickle-cell disease), HIV co-infection, malnutrition, and therapeutic immunosuppression of all types [10–15]. Approximately 5% of children with NTS infections develop life-threatening bacteremia [16]. In cohorts of African children, HIV infection is associated with a 3.2-fold increase in the odds of children presenting with invasive NTS [17]. However, the molecular mechanisms underlying the evolution of invasive and drug-resistant NTS isolates in children with co-morbidities are still unclear. The high prevalence of multi-drug resistant NTS–defined as resistance to the first line antibiotics chloramphenicol, ampicillin and cotrimoxazole–often requires use of second line antibiotics [18]. Ciprofloxacin and ceftriaxone are now recommended for treatment of invasive NTS infections [19,20]. However, resistance to both ciprofloxacin and ceftriaxone have been emerging in NTS [4,21–23].

Recent genome sequencing projects have tracked the emergence and global spread of *S*. Typhimurium and *S*. Enteritidis providing mechanistic insights into the epidemiology and evolutionary pressures of NTS [24–27]. These studies have shown that the invasive *S*. Typhimurium ST313 lineage has undergone genome degradation that appears to facilitate its invasive phenotype and highlight the rapid evolution and spread of AMR in these organisms [28–31]. However, studies also reveal that a genotypic approach to pathogen characterization does not provide a complete assessment of virulence potential or antimicrobial susceptibility [32,33]. Analyses of both genotype and phenotype have provided more accurate information regarding the capacity of a pathogen to cause invasive disease [34,35].

In this study we characterized NTS bloodstream isolates from pediatric patients hospitalized in Siaya, Kenya between 2000 and 2011 where the childhood mortality rate is ~20% in children up to 12 months old [36]. The region displays a high disease burden recording the highest prevalence of HIV (21% of adults) in Kenya in 2018 [37], 27% of children <15 years of age were infected with malaria in 2015 [38], and ~4% of them acquired an invasive NTS infection [39]. The evolutionary drivers of invasive and multi-drug resistant NTS in the presence of such co-morbidities is complicated. Here we describe both genotypic and phenotypic analysis aimed at identifying novel virulence and AMR determinants associated with invasive NTS. The outcomes of investigations like this combined with other studies supports improved antimicrobial stewardship, development of targeted countermeasures, and enhanced biosurveillance.

## Materials and methods

### Ethics statement

The National Ethical Review Committee of the Kenya Medical Research Institute and the Institutional Review Boards of the University of New Mexico (IRB CR00005628) and Los Alamos National Laboratory (IRB 13–08) approved this study. All the parents or legal guardians of the children provided written informed consent in their language of choice (Dholuo, Kiswahili or English) before enrollment into the study.

### Study area and design

Samples were collected at the Siaya County Referral Hospital (SCRH), a rural health facility in Siaya County, equatorial western Kenya, as part of an investigation to determine the burden of endemic pathogens on childhood morbidity and mortality between 2003–2009. SCRH is located in a *Plasmodium falciparum* holoendemic transmission area with increased pediatric malarial admissions, despite recent anti-malarial interventions [40]. Severe malarial anemia (SMA: hemoglobin <5.0 g/dL with any density malaria parasitemia) is the primary clinical manifestation of severe malaria in children under 5 years, peaking in children aged 7–24 months [41,42] with 53% of all the malaria-related deaths in hospitalized children under the age of 3 years due to SMA [43].

Study participants were recruited from the pediatric ward at the SCRH, Nyanza Province, western Kenya. Parents/guardians whose children (aged 3–36 months) presented at SCRH with symptoms of infectious diseases were approached for enrollment. They received an explanation of the study and HIV counseling was provided from a professionally trained counselor. Inclusion criteria included presentation at SCRH with suspected infectious disease; presence of fever ≥ 37.5°C (axillary); age 0–48 months.; parent/guardian able and willing to sign informed consent and enroll child; able to attend study appointments (over 14 days); distance to hospital ≤ 25 km. Exclusion criteria included presence of fever < 37.5°C (axillary); age > 48 months.; refusal by parent/guardian to provide informed written consent; hospitalization required for injury and/or accident (suspected non-infectious cause); parent/guardian unwilling and/or unable to attend follow-up visit (day 14); distance to hospital > 25 km. Since HIV-1 promotes anemia in children with *P*. *falciparum* malaria [44], only HIV-1 negative children were included in the present study. HIV-1 status was determined by two rapid serological antibody tests and HIV-1 proviral DNA PCR tests as previously described [44]. Children were treated according to the Ministry of Health, Kenya guidelines. All blood samples were obtained upon presentation at hospital prior to any treatment interventions.

### Bacterial cultures

Bacterial cultures were performed as previously described [39]. Briefly, blood cultures were performed for all children at enrollment in which ~1.0 mL of venipuncture blood was collected aseptically and directly inoculated into the pediatric blood culture bottle (Peds Plus, Becton-Dickinson) that were incubated in an automated BACTEC 9050 system (Becton-Dickinson), for 5 days. Positive cultures were examined by Gram staining and sub-cultured on blood agar, chocolate agar or MacConkey agar plates based on the Gram stain results. Bacterial isolates were identified according to standard microbiologic procedures as described previously [39]. According to the Kenya Ministry of Health national guideline [45], empirical antimicrobial therapy was initiated for all children with suspected bacterial infection using oral and/or injections which included combination of cloxacillin/ampicillin, chloramphenicol, ciprofloxacin/norfloxacin/nalidixic acid, ceftriaxone, gentamicin, penicillin, metronidazole or doxycycline. Children with severe malnutrition were treated with penicillin/gentamicin plus metronidazole. Antibiotic therapy was reviewed according to the results of the blood cultures.

### Antimicrobial susceptibility testing

The minimum inhibitory concentration (MIC), or lowest concentration that prevents growth, of various antibiotics was determined for *Salmonella* isolates and control organisms by disc diffusion and Etest methods [46].

*Disc Diffusion*. Antibiotic susceptibility patterns of the bacterial isolates were determined using disk diffusion (Kirby Bauer) methods performed according to the CLSI guidelines, where antibiotic resistance was defined as resistance of a microorganism to an agent to which it was previously sensitive [47]. Bacterial isolates were tested against disks of ampicillin/sulbactam (SAM, 10/10 μg), meropenem (MEM, 10 μg), piperacillin/tazobactam (TZP, 100/10 μg), cefaclor (CEC, 30 μg), cefpodoxime (CPD, 10 μg), ceftriaxone (CRO, 30 μg), ceftizoxime (ZOX, 30 μg), cefepime (FEP, 30 μg), nalidixic acid (NAL, 30 μg), ciprofloxacin (CIP, 5 μg), levofloxacin (LEV, 5 μg), azithromycin (AZM, 15 μg), clarithromycin (CLR, 15 μg), chloramphenicol (CHL, 30 μg), oxytetracycline (OTE, 30 μg), tetracycline (TET, 30 μg), and doxycycline (DOX, 30 μg). Control *Escherichia coli* and *Staphylococcus aureus* strains were run concurrently with the test organisms. Isolates with intermediate or full resistance as compared to clinical breakpoints were considered resistant (**Table 1**).

**Table 1:** Antimicrobial susceptibility testing of *Salmonella* UGA isolates by disk diffusion.

			β-lactam			cephalosporin					quinolone			macrolide			tetracycline		
					β-LI^c^	2nd	3rd generation			4th		fluoro.				-like			
Antibiotic ^a^:			**SAM**	**MEM**	**TZP**	**CEC**	**CPD**	**CRO**	**ZOX**	**FEP**	**NAL**	**CIP**	**LEV**	**AZM**	**CLR**	**CHL**	**OTE**	**TET**	**DOX**
Disk Content (μg):			10/10	10	100/10	30	10	30	30	30	30	5	5	15	15	30	30	30	30
Clinical Breakpoint (R ≤) ^b^:			11	19	17	14	17	19	21	21	13	15	13	18	ND	12	ND	11	10
	Strain ID	UGA9	7	29.5	24	23	26	30.5	31	32	17.5	31	28	12.5	7	20	18.5	17	11
		UGA10	7	33	27	25	25	33	33	32	22	33	28.5	16	7	24	23	22.5	13
		UGA11	7	27.5	23	24	26	29	33	31	25	34	31	16	8.5	7	NT	27	20
		UGA12	7	30	27	24.5	26.5	33.5	33.5	32	25	34.5	32	16.5	9	10	25	26	19.5
		UGA13	7	30	27	23.5	27	34.5	34.5	30	7	30	26	17.5	12	7	22	27	19.5
		UGA14	7	29	21	7	7	7	13	9	23.5	30	34	17	10	7	NT	10	13.5
		UGA15	18	26.5	24	21	24	26	34	29.5	22.5	35	32	15	10.5	7	NT	25	18
		UGA16	21.5	31	24	28.5	25.5	32.5	32.5	31	24	31.5	28.5	16	10	24	24	25	20
		UGA17	7	30	23.5	24	26	33	33	31.5	25	33.5	28	17	12	7	24	27	18.5
		UGA18	7	30.5	27	25	30.5	37.5	37.5	35	25	33.5	31	17	13	7	7	7	8.5
		UGA19	7	28	22	22	24.5	29	34.5	30	23.5	31.5	28	16.5	8	7	NT	26	19.5

^a^ Antimicrobial susceptibility testing performed using disk diffusion on Mueller Hinton agar: SAM, ampicillin/sulbactam; MEM, meropenem; TZP, piperacillin/tazobactam; CEC, cefaclor; CPD, cefpodoxime; CRO, ceftriaxone; ZOX, ceftizoxime; FEP, cefepime; NAL, nalidixic acid, CIP, ciprofloxacin; LEV, levofloxacin; AZM, azithromycin; CLR, clarithromycin; CHL, chloramphenicol; OTE, oxytetracycline; TET, tetracycline; DOX, doxycycline

^b^ Clinical Breakpoints reflect diameter (mm) of zones of inhibition surrounding antibiotic disk; R, resistant. Clinical Breakpoints for *Enterobacteriaceae* were derived from CLSI document M100-S23 (M02-A11) except for FEP derived from EUCAST Table v. 7.1, valid from March 13, 2017 (none listed in CLSI). No clinical breakpoints were listed in either document for OTE for *Enterobacteriaceae* (ND, not determined). AZM breakpoints are for *S*. Typhi [112].

^c^ β-lactamase inhibitor

Light grey: resistant; NT, not tested

*Etest*. *S*. Typhimurium strains ATCC 13311, UGA10 and UGA14 were grown on LB agar plates for 16–18 hours at 37°C at which point a single colony was used to inoculate 1 mL of each of the following media conditions: 1) Mueller Hinton broth (MHB, Sigma 70192); 2) MHB pH 5.5; 3) 1X M9 minimal media (Sigma M6030) supplemented with 0.1% casamino acids (Sigma 2240) and 0.3% glycerol (Sigma G5516) pH 7.4 (buffered with 100 mM Trizma base, Sigma T1503); and 4) M9 minimal media pH 5.5 (buffered with 100 mM MES hydrate). M9 minimal media was made at 1X (Sigma M6030) supplemented with 0.1% casamino acids (Sigma 2240) and 0.3% glycerol (Sigma G5516). MHB and M9 minimal media at pH 5.5 was buffered with 100 mM MES hydrate (Sigma M2933) and pH adjusted with 10% HCl (Fisher Scientific 357016). MHB is unbuffered and reaches pH 7.4 after autoclaving. M9 minimal media at pH 7.4 was buffered with 100 mM Trizma base, Sigma T1503) and pH adjusted with 10% HCl. Agar (BD Difco BD281230) for plates was added to media at 1.7%. All media was autoclaved prior to use. Overnight cultures were diluted to a 1.0 MacFarland turbidity standard (bioMérieux 70900) in 1X PBS (Sigma D8662), spread using a sterile cotton swab as instructed by the manufacturer on agar plates of each media condition, Etest strips were applied and plates were incubated at 37°C for 16–20 hours [46]. The Etest strips were purchased from bioMérieux and stored at –20°C until use: Ampicillin/Sulbactam 2/1 (412250), Ceftriaxone (412300), Ciprofloxacin (412310), Colistin (537340). Values given are mean ± standard deviation. Etests were repeated in at least 2–4 independent experiments. GraphPad Prism version 8 was used to perform a mixed-effects analysis with Tukey’s multiple comparison test to compare MICs from various media conditions to the standard antimicrobial susceptibility testing media condition of MHB (pH 7) with *P < 0.05, **P < 0.01, ***P < 0.001.

### Whole genome sequencing, assembly and annotation

The draft genomes were generated by the Los Alamos National Laboratory (LANL) Genome Science Group using Illumina technology [48]. Depth-of-coverage statistics for Illumina data are summarized in **S1 Table**. Short-insert paired-end libraries were constructed and sequenced on the HiSeq instrument. Data quality was assessed and the data files were filtered and trimmed with FaQCs, version 1.3 [49] and then assembled with Velvet, version 1.2.08 [50] and with IDBA, version 1.1.0 [51]. For UGA14, an additional PacBio [52] long read library was constructed and sequenced on the RS II instrument generating 1,869 Mbp of draft data (349X coverage), and assembled using HGAP, version 2.3.0 [53]. The consensus sequences from both short read assemblies and long read assembly (long read for UGA14 only) were computationally shredded and reassembled with Phrap, version SPS-4.24 [54,55] to create a hybrid assembly and to allow some manual editing with Consed [56]. Annotation of assembled genome sequences was carried out with the genome annotation workflow within the EDGE Bioinformatics platform [57]. Putative phase islands were identified using PHAST (PHAge Search Tool) [58] and Islandviewer [59]. The circular genome diagram was drawn using DNAplotter [60]. The whole genome sequences have been deposited in GenBank under the accession numbers listed in **Table 2**.

**Table 2:** Whole genome sequencing analysis.

Strain ID	Size	GC Content	No. of Contigs	No. of CDS	No. of RNAs	NCBI Accession No.
D23580^a^	4,996,447	51.9	2	5118	110	NC_016854, NC_013437
ATCC 13311^b^	4,831,756	52.1	2	4829	107	CP009102, CP009103
UGA9	4,940,531	52.2	40	5026	88	NHRC00000000
UGA10	5,027,081	52.2	46	5167	93	NHRB00000000
UGA11	4,951,952	52.2	44	5066	94	NHRA00000000
UGA12	4,955,481	52.2	47	5055	91	NHQZ00000000
UGA13	4,952,855	52.2	43	5051	89	NHQY00000000
UGA14^c^	5,352,626	51.9	5	5481	110	CP021462, CP021463, CP021464, CP021465, CP021466
UGA15	4,951,782	52.2	49	5060	89	NHQX00000000
UGA16	4,701,989	52.1	25	4749	96	NHTP00000000
UGA17	4,964,591	52.2	49	5074	94	NHQW00000000
UGA18	4,867,195	52.2	37	4984	85	NHTO00000000
UGA19	4,955,665	52.2	49	5068	89	NHQV00000000

^a^ including plasmid pSLT-BT (accession no. NC_013437)

^b^ including plasmid pSTY1 (accession no. CP009103)

^c^ genome completed in this work, including 4 plasmids

### Genome comparison

All closely related NTS isolates were first identified using megablast searching against GenBank and were further compared with the related *S*. Typhimurium strains D23580, ST313, ATCC 13311, and *S*. Enteritidis CMCC 50041. To obtain a list of orthologs from bacterial genomes, an open source protocol that determines bidirectional best hits was used [61]. The process considers orthologs as genes *g* and *h*, if *h* is the best BLASTP hit for *g* and *vice versa*, and E values were ≤10–15. A gene is considered strain specific if it has no hits with an E value of 10–5 or less. Browsing of genome comparisons at the nucleotide level was carried out using the Artemis Comparison Tool (ACT) [62].

PhaME version 1.0.5 [63] was used to generate the genome alignments, extract all SNPs at conserved positions, and infer the phylogenetic tree. Briefly, raw reads were mapped to *S*. *enterica* subsp. enterica serovar Typhimurium str. SL1344 (NC_016810.1) [64] using BWA v0.7.17-r1188, followed by construction of alignment, removal of polymorphic sites using gubbins v2.4.1, and then reconstruction of maximum likelihood phylogenetic tree with 100 bootstraps using IQ-TREE v1.6.12. The built-in model test of IQ-TREE was invoked using *-m TEST* option, which picked K3P+ASC as the best fit model.

Treefiles were annotated with iTOL v4 to include strain ID numbers, bootstrapping symbols, and tree scale as substitutions per variable site [65]. The resulting file was further edited in Adobe Illustrator to discriminate lineages, and add features such as a legend, geographical locations, and respective references.

### Phenotype microarray analysis

*S*. Typhimurium strains ATCC 13311, UGA10 and UGA14 were grown on nutrient agar plates for 18 hours at 37°C. A single colony swab of culture from each strain was re-suspended in the appropriate medium for each plate and adjusted to 85% transmittance measured using a Biolog turbidimeter. The recommended inoculating fluids, IF-0a and IF-10a were used with Biolog Redox Dye D. These cultures were diluted 1:100 in the appropriate medium and Biolog phenotypic microarray plates PM1 through PM20 were inoculated with 100 μL per well. Plates were incubated at the 37°C in the OmniLog incubator and substrate utilization/chemical sensitivity was monitored every 15 minutes for 48 hours. Bacterial respiration was assessed within each well by monitoring color formation resulting from reduction of the tetrazolium dye, and color intensity was expressed in arbitrary units (AU). Kinetic data was analyzed using Omnilog-PM software (OL_PM_Par1.20.02, Dec. 08, 2005).

### Statistical analyses and heatmap generation

The area under the growth curves (AUC) were computed by summing all OmniLog values at all time points using the opm package and the R programming language [66]. AUC values of selected phenotypes were plotted in a heatmap using the Morpheus tool (https://software.broadinstitute.org/morpheus). High growth (AUC) was represented by yellow blocks while blue blocks represented low growth.

### Membrane permeability

The membrane permeability of *S*. Typhimurium ATCC 13311, UGA10 and UGA14 isolates was determined using the BacLight Bacterial Membrane Potential Kit as per the manufacturer instructions (Life Technologies, catalog no. B34950). Briefly, bacterial strains were grown overnight at 37°C shaking in nutrient broth. 1 μL of stationary phase culture was transferred to 1 mL of sterile-filtered phosphate buffered saline (PBS). Then 10 μL of the DiOC_2_(3) dye was added, as recommended by the manufacturer. The assay was verified using the protonophore carbonyl cyanide m-chlorophenyl hydrazone (CCCP). Cells were incubated with dye for 30 min at room temperature, and then analyzed on a BD FACSAria flow cytometer (Becton Dickinson), with emission filters suitable for detecting red (PE-Texas Red) and green (FITC) fluorescence. Ten thousand events were recorded at a medium flow rate. Gating of stained cell population and analysis of flow cytometry data were performed in BD FACSDiva software (Becton Dickinson). The ratio of red to green fluorescence intensity was calculated as an indicator of membrane potential. Values given are the mean of nine biological replicates ± standard error of the mean. GraphPad Prism version 8 was used to perform a one-way ANOVA with Dunnett’s multiple comparison test to compare ATCC 13311 + CCCP, UGA10 and UGA14 to ATCC 13311 (***P < 0.001).

### Zeta potential measurements

Overnight cultures were started as described in *Etest* section above. Since media composition (salt concentration and pH) can affect zeta potential, [67], 0.5 mL of each culture was washed in 1X PBS and resuspended in 1 mL 0.1X PBS diluted in sterile nanopure water to minimize media influence on these measurements. We also report conductivity measurements to show that salt concentration has been normalized by this procedure. The Zeta potential was measured on a Malvern Zetasizer Nano ZS90 device (Malvern) at room temperature 25°C. Zeta potential standards were obtained from Malvern (DTS1235, –42 mV ± 4.2 mV) and used according to the manufacturer’s instructions. Measurements were performed in Zetasizer folded capillary cells (DTS1070, Malvern) in triplicate reads (n = 3). Values given are mean ± standard deviation. Zeta potential assays were repeated on at least two separate occasions. GraphPad Prism version 8 was used to perform a two-way ANOVA with Tukey’s multiple comparison test to compare UGA strains 10 and 14 to the antimicrobial susceptible strain ATCC 13311 (*P < 0.05, **P < 0.01, ***P < 0.001).

## Results

### Genotypic analysis

**Fig 1:**
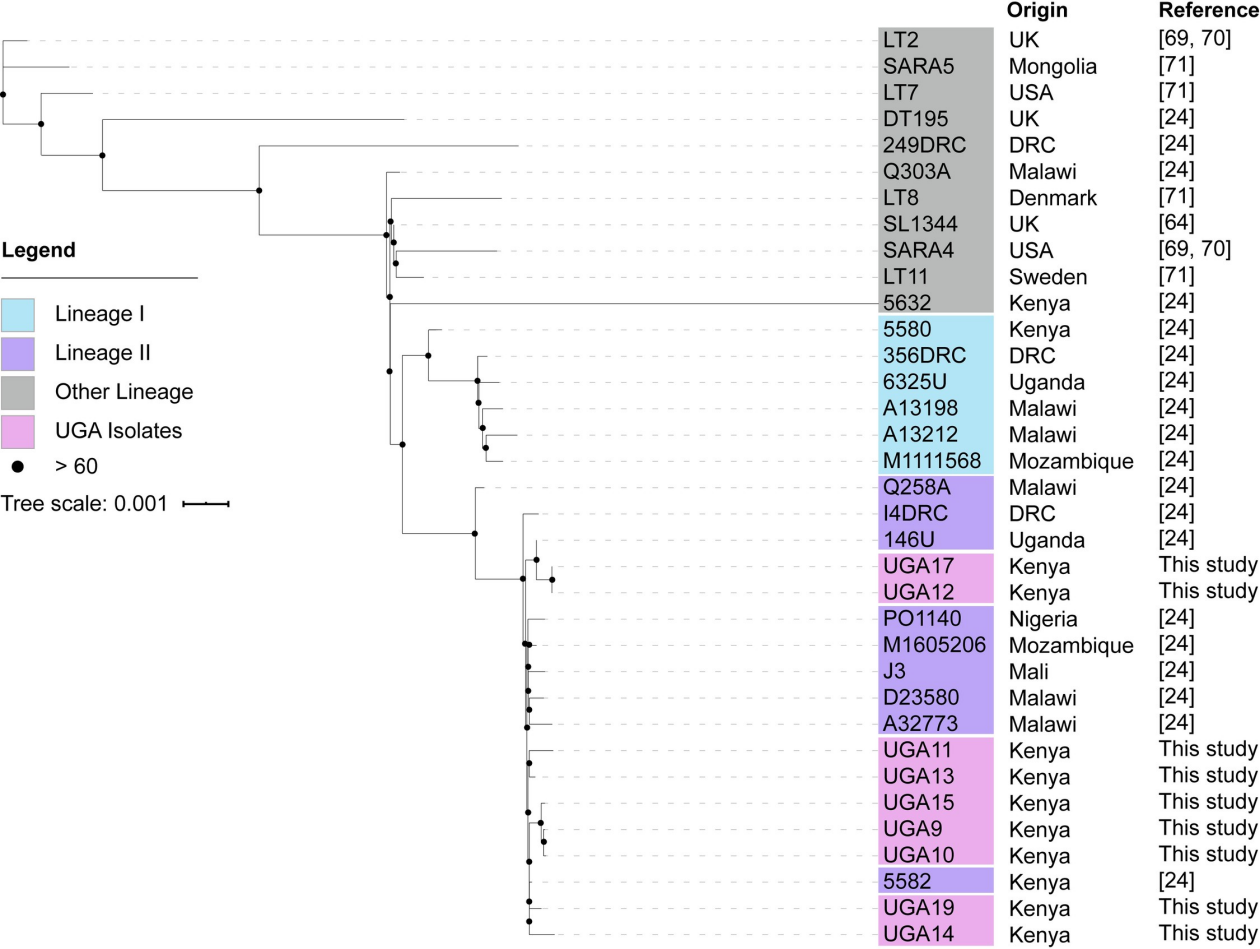
A maximum likelihood single-nucleotide polymorphism (SNP)–based phylogeny of *S*. Typhimurium isolates from Kenya between 2000 and 2011 associated with invasive disease. The strains that were sequenced as part of this study are represented as UGA followed by a number, and all other strains were previously reported by the publications indicated in the “Reference” column. Names of genomes from this study are colored in pink, isolates from lineage 1 and lineage 2 identified previously by Okoro *et al*. [24] are colored in cyan and purple respectively, and all other genomes are colored in gray. The tree was rooted using the midpoint method in iTOL. The scale bar indicates number of substitutions per site. Bootstrap support are labeled on branches with black dots whose size correspond to bootstrap support values.

**Fig 2:**
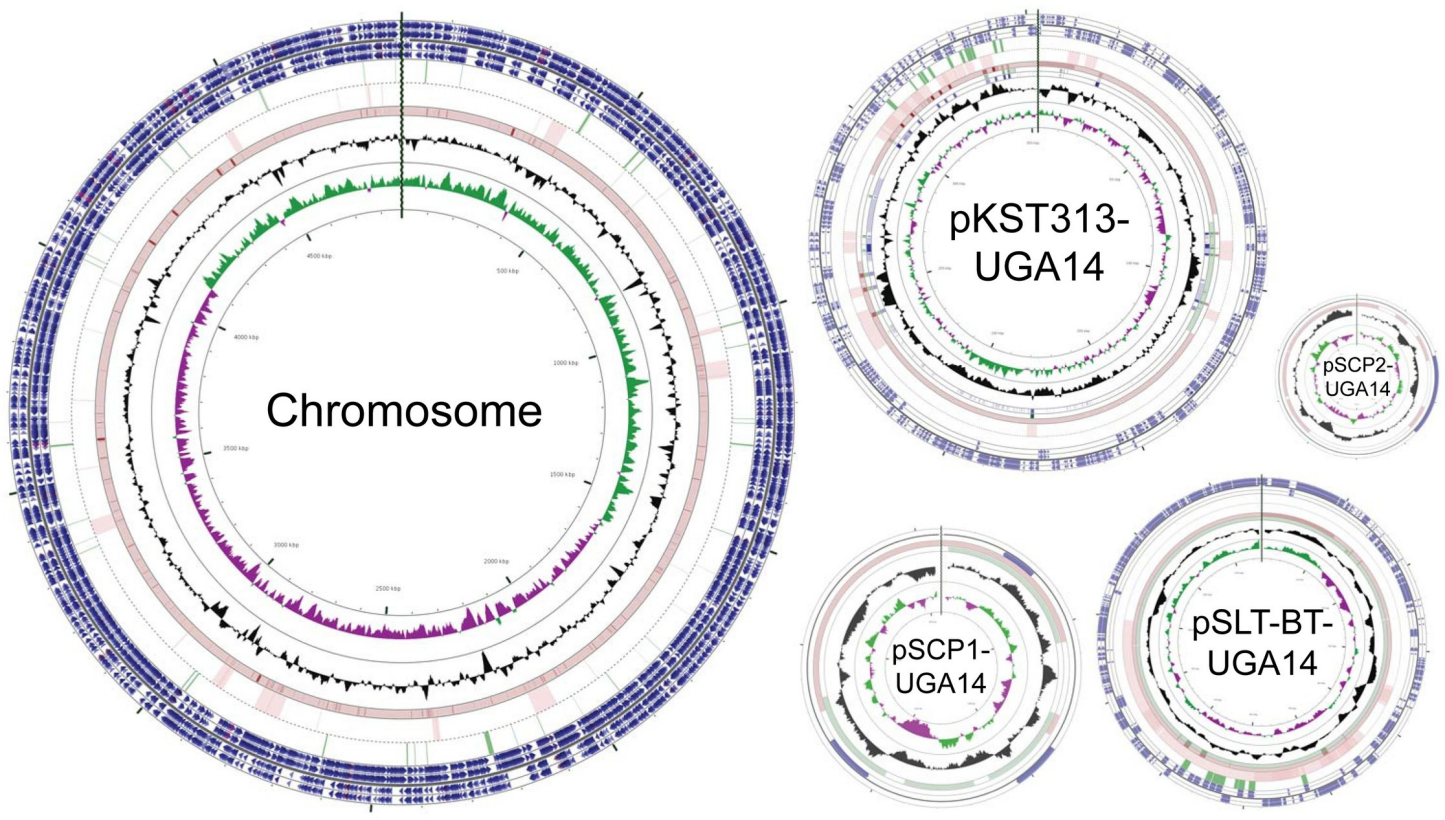
Circular representation of the *S*. Typhimurium UGA14 genome including the chromosome and four plasmids. The outermost four circles indicate start sites of genes. Starting on the outside, circles 1–2 consist of forward-strand gene products; circles 3–4 consist of reverse-strand gene products (colors represent the following categories: CDS, blue; tRNA, brown; rRNA; magenta; other, grey); circle 5 shows AMR genes; circle 6 shows mobile gene elements (insertion sequences and prophage genes); circles 7–9 show homologous regions of blastn search against near neighbors in GenBank (highly similar regions are shown in darker color); circle 8 shows GC content; circle 9 shows GC bias (G-C/G+C where green indicates values >1 and purple <1).

We obtained 11 bloodstream NTS isolates from pediatric malaria patients in Siaya, Kenya that were isolated as a part of a comorbidity study described previously (**Table 3**) [39,68]. Initial whole genome sequencing was performed using Illumina technology on all 11 isolates, which identified nine *S*. Typhimurium (labeled UGA9, 10, 11, 12, 13, 14, 15, 17, 19) and two *S*. Enteritidis (UGA16, 18) isolates with a depth of sequencing typically greater than 300X (**Table 2 and S1 Table**). Phylogenetic analyses based on whole-genome single-nucleotide polymorphisms (SNPs) was performed to compare these isolates to those described in previous studies by others (**Fig 1** and **S2 Table**). The SNP tree in **Fig 1** compares the UGA isolates identified in this study with invasive isolates from other studies [69–71]. The nine *S*. Typhimurium UGA strains in this study all belong to lineage II showing only 30 SNPs between UGA14 and the D23580 strain isolated from a bloodstream infection in Malawi in 2004 [28]. This observation is consistent with previous findings that lineage II strains replaced the previously dominant lineage I strains across Africa [28,72]. In order to accurately reconstruct the genome across long repetitive stretches, *S*. Typhimurium UGA14 was supplemented with long-read PacBio RS technologies. After hybrid assembly, we obtained a complete genome of 5,352,626 bp in size with a 4.9 Mbp chromosome, two large plasmids and two small plasmids (**Fig 2** and **Table 4**). The genome size and presence of four plasmids is consistent with that of D23580 [28,73]. However, despite the nearly identical chromosome, there were significant differences in the plasmid composition.

**Table 3:** Non-typhoidal *Salmonella* clinical isolates.

Strain ID	Isolate #	Serovar
UGA9	022-04B	Typhimurium
UGA10	224–09	Typhimurium
UGA11	366–04	Typhimurium
UGA12	159-04B	Typhimurium
UGA13	376–09	Typhimurium
UGA14	709–09	Typhimurium
UGA15	547–09	Typhimurium
UGA16	215–09	Enteritidis
UGA17	159-04A	Typhimurium
UGA18	271–09	Enteritidis
UGA19	654–05	Typhimurium

**Table 4:** UGA14 plasmid characteristics.

ID	Plasmid Type	Near Neighbor	Length (bp)
pKST313-UGA14	*IncHI2*, *IncHI2A*	pKST313 (accession no. LN794248)	352,906
pSLT-BT-UGA14	*IncFII(S)*, *IncFIB(S)*	pSLT-BT (117047bp)	115,864
pSCP1-UGA14	SCP	pSA01AB09084001_4; pPAB19-3	2,576
pSCP2-UGA14	SCP	pRGRH0639	1,995

SCP: small cryptic plasmid

### Plasmids

**Fig 3:**
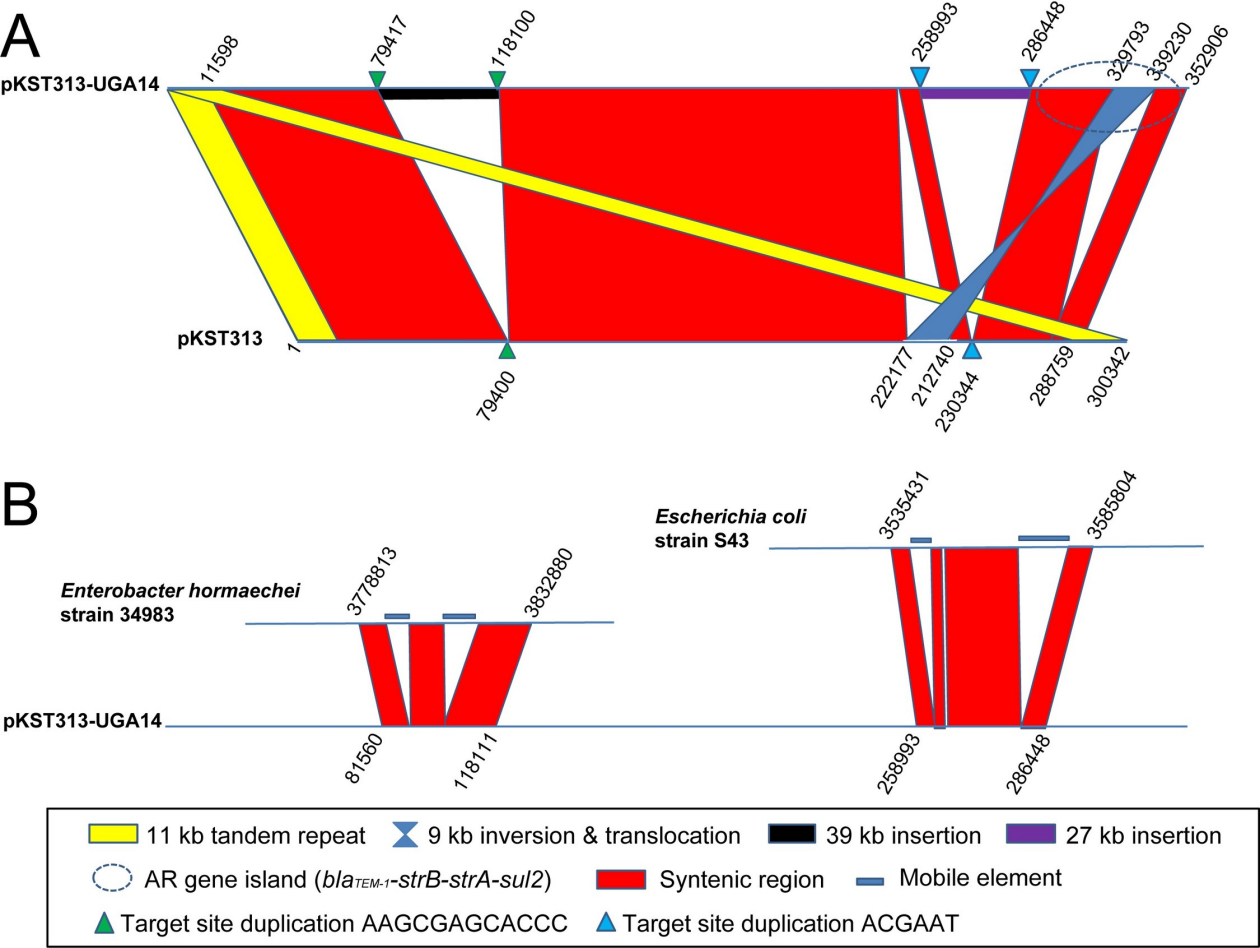
Plasmid synteny between *S*. Typhimurium UGA14 plasmid pKST313-UGA14 and reference plasmids. Comparisons were made using the Artemis Comparison Tool (ACT) [62]. (A) pKST313-UGA14 compared to reference plasmid pKST313 [21] with regions of homology indicated in red or blue (inversion), an AMR gene island indicated with a blue circle (dashed line), direct repeats in pKST313 highlighted in yellow, and loci of insertion and its target site duplication labeled with triangles. (B) Two inserted regions of pKST313-UGA14 are compared with potential donor organisms, *Enterobacter hormaechei* strain 34983 and *Escherichia coli* strain S43.

The largest plasmid identified in UGA14 was similar (81% query coverage, 99% sequence identity) to the pKST313 plasmid identified by Kariuki *et al*. [21] except that it was ~50 kb larger in size at 352,906 bp and referred to as pKST313-UGA14 (**Table 4**). This plasmid was determined to be very similar to the pKST313. In comparison with pKST313, pKST313-UGA14 was found to contain two large gene cluster insertions (**Fig 3**). The first insertion of 39 kb is homologous to the chromosome of *Enterobacter hormaechei* subsp. *hormaechei* strain 34983 with 94% coverage and 99% sequence identity, containing an intact *lac* operon and a complete iron(III) transporter system. The second insertion of 27 kb appears to be derived from the chromosome of *Escherichia coli* strain S43 with 70% coverage and 99% sequence identity, containing enzymes involved in sucrose metabolism. pKST313-UGA14 also contained an inversion/translocation of 9 kb flanked by mobile elements. This region harbors a *bla*_TEM-1_-*strB-strA-sul2* AMR gene cluster, which encodes resistance to penicillins, cephalosporins and aminoglycosides [21]. Interestingly, this plasmid was not detected in any of the other UGA isolates sequenced in this study.

The 115,864 bp pSLT-BT plasmid identified in UGA14 (referred to as pSLT-BT-UGA14) is a known virulence-associated plasmid essential to systemic invasiveness of *S*. Typhimurium (**Table 4** and **S1 Fig**) [74]. Comparison of pSLT-BT-UGA14 with the sequences of pSBLT (ST313) [21] and pSLT-BT (D23580) [28,73] reveals extensive overall similarity with the exception of a 19 kb inversion (**S1 Fig**). This plasmid was observed in all nine *S*. Typhimurium UGA isolates (**S3 Table**). All previously identified resistance determinants associated with this plasmid were identified− aminoglycoside (*aadA1*), streptomycin (*strA* and *strB*), β-lactams (*bla*_TEM-1_), chloramphenicol (*catA1*), trimethoprim (*dhfr1*), and sulfonamides (*sul1* and *sul2*) [21,28,73,74]. A comparison of UGA14 plasmids pKST313-UGA14 and pSLT-BT-UGA14 shows extensive transfer of virulence and AMR related genes (**S2 Fig**). The two insertion regions of pKST313-UGA14 appear to be conserved with an *Enterobacter* chromosome background along with mobile gene elements at the flanking regions of both the lactose and sucrose operons all suggest the acquisition of these regions through horizontal gene transfer (HGT, **Fig 3B**) [75]. The corresponding region in pSLT-BT-UGA14 (47,894 to 69,936 bp) is also located at the flanking region of the *strB-strA-sul2* cluster (**S2 Fig**, grey arrows) and is inverted relative to pSLT-BT.

The small plasmids (referred to as pSCP1-UGA14 and pSCP2-UGA14) were identified as small cryptic plasmids (SCPs), which are a class of unusually small, abundant plasmids that carry little genetic information yet inexplicably maintained at high copy numbers [76]. These small plasmids were very similar to those identified in D23580, which contained plasmids pBT2 (2,556 bp) and pBT3 (1,975 bp) [28,73]. UGA14 contained plasmids pSCP1-UGA14 (2,576 bp) and pSCP2-UGA14 (1,995 bps) as shown in **Table 4**. Plasmid pSCP1-UGA14 was determined to be most similar to pPAB19-3 from *Escherichia coli* strain M9888 with 68% coverage and 92% sequence identity (**S3 Fig**). Plasmid pSCP2-UGA14 showed no homolog among any other *Enterobacterales* in the NCBI NR database and aligned with an uncultured prokaryote isolate RGRH0639 from a rat gut metagenome with 72% coverage and 92% sequence identity (**S4 Fig**). P4 only carries a hypothetical protein and MarR, which is a repressor of the multiple antibiotic resistance (*mar*) operon [77]. The *mar* regulon mediates AMR by activating AcrAB-TolC efflux of some antibiotics, disinfectants and organic solvents, and down regulating influx through Outer Membrane Protein F (OmpF) [78]. The two SCPs (pSCP1-UGA14 and pSCP2-UGA14) were identified together in UGA11, 12, 13, 17, and 19 while pSCP2-UGA14 alone was found in UGA9 and UGA10 (**S3 Table**). Interestingly, UGA15 was the only *S*. Typhimurium isolate sequenced that did not contain a homolog of either of these two SCPs.

In the two *S*. Enteritidis isolates, UGA16 harbors a smaller pSENV plasmid (~60 kb) and UGA18 harbors two larger plasmids: pSEN-BT (termed pSEN-BT-Siaya, ~85 kb) and p931 (p931-UGA14, ~93 kb). Seven AMR genes were found on the UGA18 pSEN-BT-Siaya. This observation is similar to the one made in the *S*. Enteritidis strain D7795 wherein pSEN-BT was composed of a backbone of pSENV with regions that harbor 9 antibiotic resistance genes and additional genes associated with virulence and toxin/antitoxin induction systems [79]. Interestingly, UGA18 plasmid p931-UGA14 is very similar to the *S*. Typhimurium UGA10 plasmid pSLT-BT-UGA14 with 90% coverage and 98% sequence identity (**S5 Fig**).

### Genome degradation

It has been previously reported that *S*. Typhimurium strain D23580 has undergone reductive evolution through pseudogene formation [28,80]. We compared the pseudogene content of strain UGA14 with D23580 and identified 44 pseudogenes that had previously been reported in D23580 [28,73]. Interestingly, the *macB* (STM0942) gene encoding macrolide resistance [81] was found to be disrupted in D23580 but intact in UGA14. We also identified three pseudogenes in UGA14, all resulting from point mutations encoding early stop codons, that remained intact in D23580. These occurred in *nfsA* (NADPH nitroreductase), *dgoA* (D-galactarate dehydratase) and *ccmA* (cytochrome c biogenesis ATP-binding export protein). Missense mutations in *nfsA* have been associated with nitrofurantoin resistance in *S*. Typhimurium [82]. Inactivation of *dgoA* in *S*. Typhimurium impacts galactonate metabolism [83], and mutations in *ccmA* affect cytochrome biosynthesis in *E*. *coli* [84]. We also confirmed the presence of identical mutations in UGA14 that were previously identified in D23580 including *rpoS*, *katE*, *bcsG*, and *sseI* [28,73]. Inactivating mutations in genes such *rpoS* have been observed in *S*. Typhimurium following laboratory passaged in minimal media [85]. However, mutations in *macB*, *nfsA*, *dgoA* and *ccmA* have not been observed in *S*. Typhimurium following passage in LB, Mueller Hinton Broth (MHB) or minimal media conditions. Also, the *macB*, *nfsA*, *dgoA* and *ccmA* mutations were not observed in UGA10, which was grown in the same conditions as UGA14.

### Phenotypic analysis

To complement the genotypic analysis, we performed phenotypic characterization of the NTS isolates in this study to evaluate changes in their ability to grow in various environmental conditions relative to a non-invasive and drug-sensitive reference *S*. Typhimurium strain ATCC 13311 [86].

### Antimicrobial resistance

**Fig 4:**
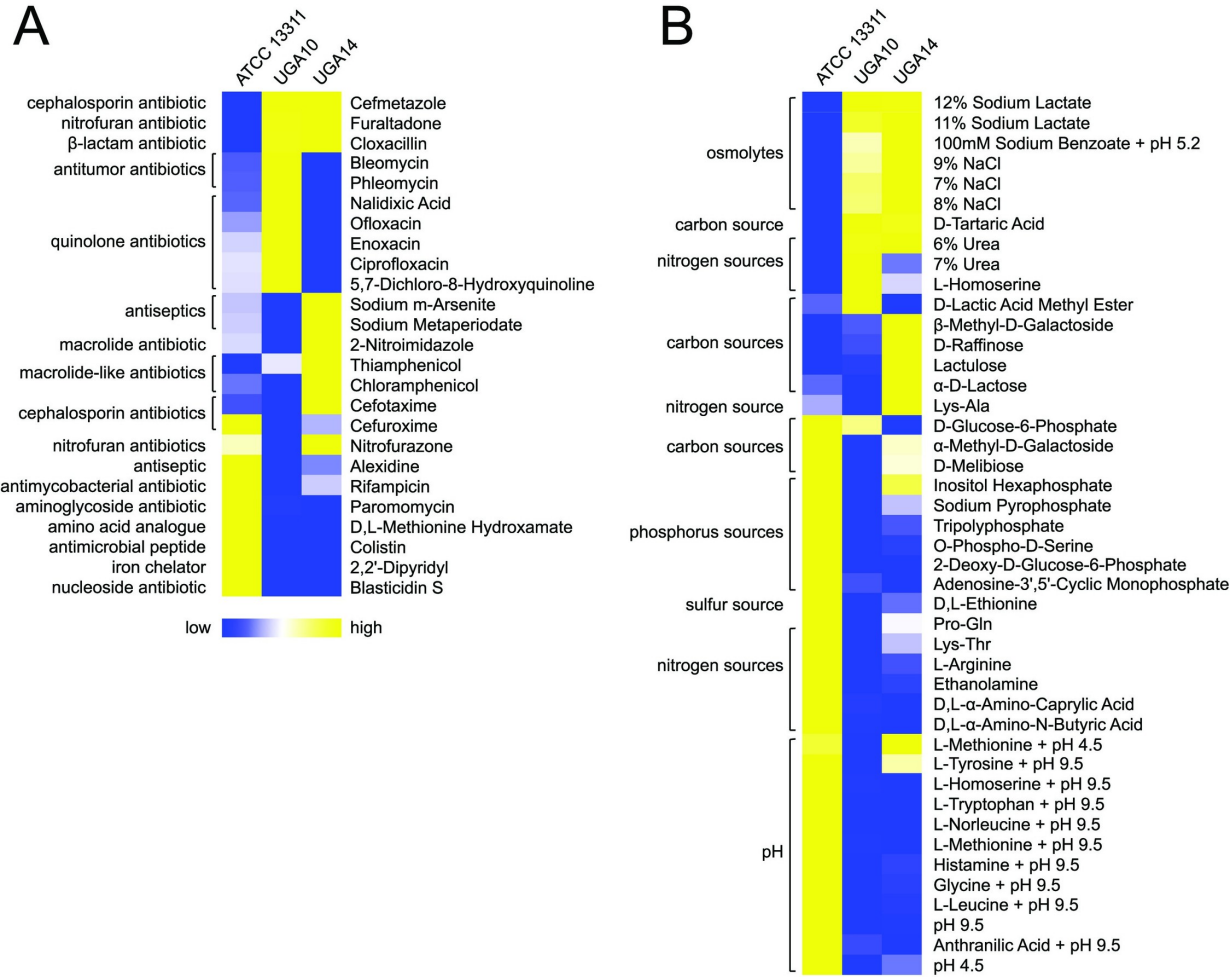
Heatmaps of selected Biolog phenotypic array profiles for *S*. Typhimurium strains. Data are grouped into categories based on their biological roles as either (A) chemicals and antimicrobials or (B) nutrients. *S*. Typhimurium reference strain ATCC 13311 (antibiotic susceptible and non-invasive) was compared to isolates UGA10 and UGA14. Each column represents the area under the curve (AUC) values computed by adding all OmniLog values at all time points. All raw AUC data is available in **S1 Data**. Blue represents low relative growth in a given condition while yellow represents high growth.

NTS isolates from Kenya have displayed resistance to ampicillin, chloramphenicol, sulfamethoxazole-trimethoprim, and more recently cephalosporins [21,28]. The antibiotic susceptibility patterns for the 11 NTS isolates described in this study were determined using disk diffusion according to the Clinical Laboratory Standards Institute (CLSI) guidelines and antibiotic resistance was defined by clinical breakpoints (**Table 1**) [47]. *S*. Typhimurium isolates UGA10 and UGA14 were further characterized using Biolog phenotypic arrays [87] to validate antimicrobial resistance changes as compared to ATCC 13311 (**Fig 4**). 9 out of 11 UGA isolates were resistant to ampicillin/sulbactam, however, all 11 remained susceptible to meropenem and piperacillin/tazobactam. Biolog experiments confirmed these observations (**Fig 4A** and **S1 Data**). All ampicillin-resistant UGA isolates have a homolog of glyoxylase or a related metal-dependent hydrolase, belonging to the β-lactamase superfamily II, on their chromosome.

Chloramphenicol resistance was observed in 8 of 11 UGA isolates (not UGA9, 10, or 16). Genome comparison shows UGA11, 12, 13, 15, 17, and 19 each have one copy of *catA1* on plasmid pSLT-BT-UGA14, while UGA18 has one copy of *catA2*. UGA14 has 3 *cat* genes with *catB3* and *catA1*/*catI* located on pKST313-UGA14, and another copy of *catA1* on pSLT-BT-UGA14. In contrast, the chloramphenicol susceptible isolates UGA9, 10, and 16, all lack the *catA1/catI* and *catB3* genes. Biolog results confirmed these findings showing a 2- to 3-fold increase in cellular respiration by UGA14 in the presence of thiamphenicol (an analogue of chloramphenicol), compared to the chloramphenicol-sensitive *S*. Typhimurium ATCC 13311 and UGA10 strains (**Fig 4A** and **S1 Data**).

The *sul1* and *sul2* genes encoding sulfonamide resistance were found in 9 out of 10 UGA isolates (only absent from *S*. Enteritidis UGA16) although phenotypic assessments of resistance were not performed. Cephalosporin resistance was only observed in UGA14, which can be explained by the presence of *bla*_*CTX-M-15*_, a type A β-lactamase on pKST313-UGA14 as described previously by Kariuki *et al*. [21]. UGA13 was uniquely resistant to nalidixic acid as a result of two substitutions, D87G and D94G, found in the chromosomal DNA gyrase protein GyrA which is the target of quinolone antibiotics [88]. *S*. Typhimurium isolate UGA14 and *S*. Enteritidis isolate UGA18 were the only two strains to display tetracycline resistance each containing a plasmid-derived *tet* gene.

Additionally, there were AMR phenotypes that were not readily explained by the genotypic analysis. Ampicillin/sulbactam resistance was observed in UGA18, but not in UGA16, when grown in standard antimicrobial susceptibility testing conditions (MHB) despite both containing the same gene profile of β-lactamases. No inducible resistance was observed following growth in any of the media conditions tested (**S6A Fig**). UGA14 contains the pKST313-UGA14 mediated *aac(6')-Ib-cr* gene encoding fluoroquinolone resistance, but no corresponding resistance was observed when tested by disk diffusion (ciprofloxacin and levofloxacin in **Table 1**). However, in Biolog arrays UGA14 displayed low-level resistance to ciprofloxacin and lomefloxacin compared to the ATCC 13311 reference strain (**S1 Data**). Interestingly, UGA10 which does not carry the *aac(6')-Ib-cr* gene, outgrows UGA14 under the highest concentration of ciprofloxacin and lomefloxacin. When tested under various growth conditions, increased resistance to ciprofloxacin was observed at pH 7 when compared to pH 5.5 for all three strains tested (**S6B Fig**), which has been observed previously [32]. Together, these results highlight a discrepancy between genotypic and phenotypic observations that could impact the treatment efficacy and outcome of invasive NTS infection.

### Heavy metal resistance

The pKST313 plasmid identified previously is known to contain genes involved in resistance to heavy metals including mercury (*mer* and *tni*), tellurite (*ter*), arsenic (*ars*), and copper (*cusS* and *pcoE*) [21]. The pKST313-UGA14 found in UGA14 similarly contains the heavy metal resistance genes for arsenic (*ars*), mercury (*mer*), copper (*cusS*) and tellurite utilization (*tel*). Biolog data confirmed several of these phenotypes. UGA14 displays a 2.4-fold increase in resistance to sodium *m*-arsenite over the *S*. Typhimurium ATCC 13311 reference strain, and a 5.5-fold increase in resistance over the sensitive UGA10 isolate (**Fig 4A** and **S1 Data**). Genes encoding the tellurite resistance proteins TehB and TehA are found on pKST313-UGA14 as well as on the chromosomes of UGA14, UGA10, and ATCC 13311. This was confirmed phenotypically in the Biolog experiments where all 3 strains display similar metabolic patterns in the presence of potassium tellurite (**S1 Data**).

### Membrane permeability

**Fig 5:**
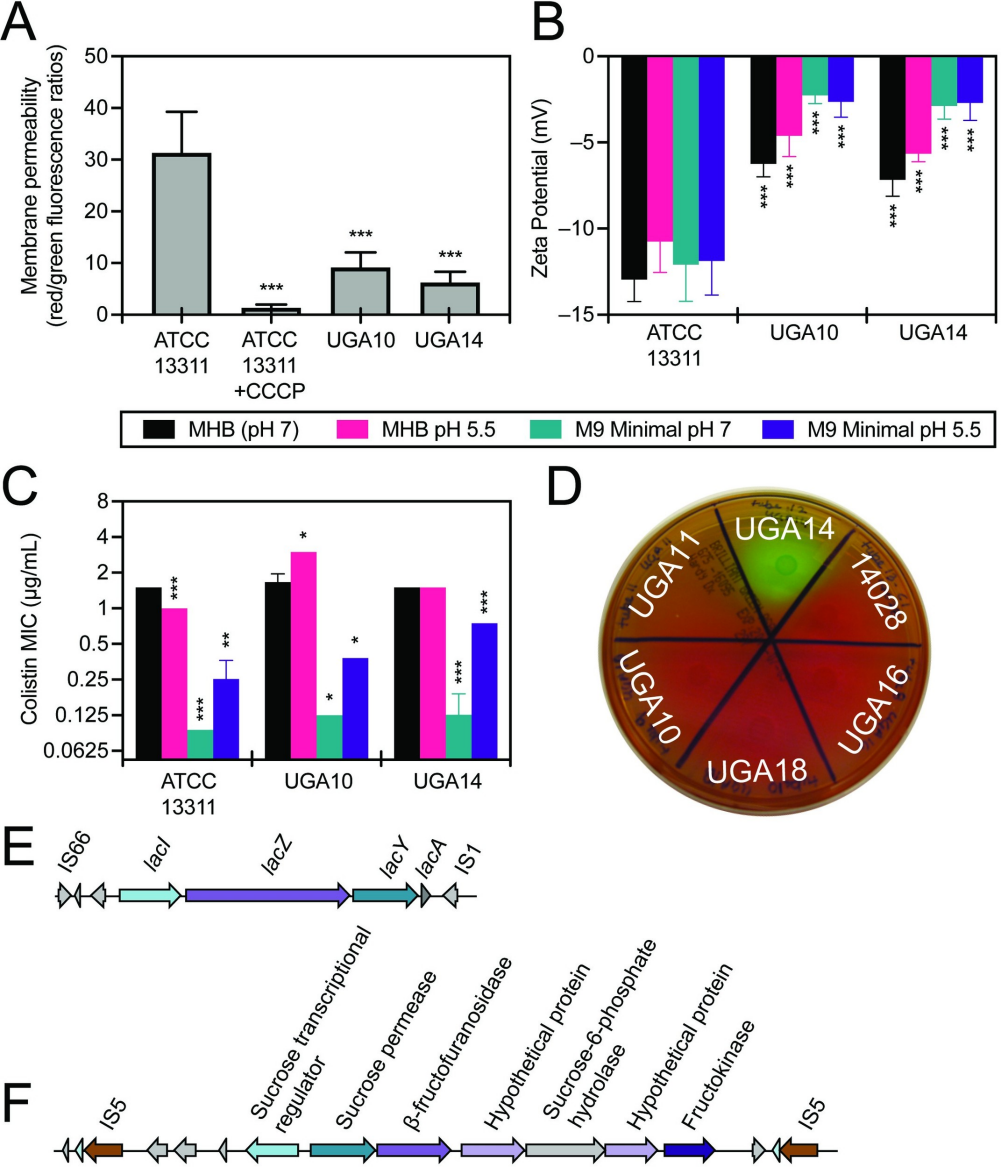
*S*. Typhimurium isolates UGA14 and UGA10 displayed altered membrane permeability and zeta potential compared to the antimicrobial sensitive and non-invasive ATCC 13311 strain. Both UGA14 and UGA10 displayed reduced (A) membrane permeability and a (B) more positive zeta potential compared to the reference strain ATCC 13311. Alterations in zeta potential were independent of growth conditions comprised of either Mueller Hinton Broth (MHB, unaltered at pH 7) or minimal media made with M9 salts supplemented as described in the Materials and Methods section (M9 Minimal) adjusted to either pH 5.5 or 7. (C) The minimum inhibitory concentration (MIC) of colistin is significantly reduced in minimal media conditions as compared to standard MHB susceptibility testing conditions. Uniquely for a *Salmonella* strain, UGA14 (D) displays growth on brilliant green agar indicating fermentation of lactose and/or sucrose, (E) contains an intact *lac* operon, and (F) contains a plasmid-mediated sucrose regulon. *S*. Typhimurium ATCC 14028 (labeled “14028” in (D)) is a highly virulent reference strain [113] that lacks the ability to ferment lactose and sucrose. Values plotted are mean ± standard deviation. Membrane permeability represent the average of nine independent experiments each recording 10,000 events. Zeta potential represent the average of triplicates from three independent experiments. MIC determinations represent the average of 2–4 independent experiments. Statistical significance was determined for UGA10 and UGA14 values compared to the reference strain ATCC 13311 with *P < 0.05, **P < 0.01 and ***P < 0.001.

Biolog experiments revealed that UGA14 and UGA10 were highly resistant to ionic solutions (osmolytes) as compared to the non-invasive reference *S*. Typhimurium ATCC 13311 strain (**Fig 4B**). In order to determine if this phenotype was related to alterations in membrane permeability, we directly analyzed the membrane potential of these isolates using the BacLight bacterial membrane potential kit (Invitrogen). In this assay, the dye diethyloxacarbocyanine (DiOC_2_(3)) emits green fluorescence when outside of a cell but aggregates once inside causing a shift to red emission [89]. Measurements are taken by flow cytometry on the same number of bacterial cells in a population, and the ratio of red to green fluorescence is determined as an indicator of permeability of the bacterial membrane. These experiments showed that UGA10 and UGA14 display a 3- to 5-fold reduction in membrane permeability as compared to the reference ATCC 13311 strain (**Fig 5A**). UGA10 and UGA14 also displayed a more positive surface charge than ATCC 13311 as measured by zeta potential (**Fig 5B**). The zeta potential of antimicrobial sensitive *S*. Typhimurium strain ATCC 13311 was –12.96 ± 1.27 mV in MHB, which is consistent with previous reports [90]. UGA10 and UGA14 displayed significantly more positive zeta potentials of –6.25 ± 0.75 mV and –7.17 ± 0.94 mV respectively (*P <* 0.001). Reduced membrane permeability has been linked to antimicrobial resistance [91] as well as resistance to cationic antimicrobial peptides [92]. When grown in minimal media conditions at pH 7, like the conditions reflected in Biolog experiments, UGA14 and UGA10 were highly sensitive to the cationic antimicrobial peptide colistin as compared to the reference strain ATCC 13311 (**Figs 4A** and **5C**). However, when grown in MHB (both pH 7 and 5.5) as well as M9 minimal conditions at pH 5.5, we observed a significant increase in MIC to colistin for both UGA10 and UGA14. This was interesting considering that the surface charge of these isolates was not significantly different following growth in M9 minimal media at pH 7 compared to pH 5.5 (**Fig 5B**).

### Nutrient utilization

UGA14 has the rare ability for a *S*. Typhimurium isolate to metabolize lactose and sucrose. This was identified using Biolog phenotypic arrays (**Fig 4B** and **S1 Data**) and confirmed by growth on brilliant green agar (**Fig 5D**). Brilliant green agar is often used to easily differentiate *Salmonella* from *E*. *coli* since the fermentation of either lactose or sucrose will produce acid causing the media to turn yellow/green [93]. Lactose fermentation is rarely observed in *Salmonella* since the presence of the *lac* operon has been shown to inhibit epithelial cell invasion by repressing flagellar biosynthesis [94]. Similarly, sucrose utilization is observed in less than 10% of *Salmonella* strains [95]. Therefore, classically NTS produce white or red colonies on brilliant green agar while *E*. *coli* produce yellow/green colonies making their classification distinct. Genotypic analysis confirmed the presence of an intact *lac* operon as well as a sucrose catabolism (*csc*) regulon on pKST313-UGA14 (**Fig 5E** and **5F**). These results indicate that traditional microbiological methods for identifying and characterizing NTS isolates from patients may misdiagnose them as *E*. *coli*.

### Discussion

Invasive NTS are among the leading cause of bloodstream infections in sub-Saharan Africa, especially in high risk groups such as children and HIV-infected individuals [96]. The spread of invasive *S*. Typhimurium across Africa has been documented in Ghana, Burkina Faso, and Guinea-Bissau [97], Kenya [21,28,98], Gambia [99] the Democratic Republic of the Congo (DRC) [22,23,100], Nigeria [100], and Malawi [11,28] with the phylogeography of the spread presented by Okoro *et al*. [24]. Emergence and spread of invasive *Salmonella* has been demonstrated globally for *S*. Typhi, which has been shown to be a highly clonal evolutionary path [101,102]. Major concerns with the global spread and evolution of NTS include the pervasive invasiveness and increasing AMR observed among isolates.

In this study, we have taken an integrative approach to compare both genotypic and phenotypic information in clinical samples that caused invasive disease in children in Siaya, Kenya. Here, 9 of 11 isolates were identified as *S*. Typhimurium ST313, a newly emerging serovar associated with invasive disease [33]. Work from other investigators clearly shows that ST313 is becoming human-adapted, losing the ability to utilize some common carbon sources, and acquiring AMR [21,28–31,98,103]. The existence of pseudogene heterogeneity among *S*. Typhimurium is indicative of genome decay which has been observed as pathogens adapt to their new hosts.

These observations are consistent with the hypothesis that *Salmonella* evolved through several phases due to acquisition of additional pathogenicity elements through HGT [104]. Acquisition of the pKST313 plasmid in NTS isolates has been shown to result in the development of resistance to third-line antimicrobials [21]. The pKST313-UGA14 identified in this study now contains lactose and sucrose utilization genes obtained through HGT. Lactose-fermenting *Salmonella* isolates are difficult to identify as NTS and failure to detect them is a threat to both human and animal health [105]. The continued evolution of the pKST313 plasmid is also evident in the AMR island shared between pKST313-UGA14 and pSLT-BT-UGA14. A wide range of AMR genes are represented in this duplication showing that numerous pathways exist for HGT of these genes across many bacteria in a given geographic area. Both AMR genes and virulence factors can propagate by these mechanisms, which can result in the emergence of invasive pathogens against which our current intervention strategies are not effective [106,107].

All of the isolates we examined contained one or more plasmids with β-lactamase genes. Of these isolates, those with a β-lactamase on the chromosome predominately contained a penicillin-binding protein (PBP). Isolates with a β-lactamase on a plasmid contained a class A β-lactamase associated with broad-spectrum resistance. *S*. Typhimurium strain UGA14 contained a multiple β-lactamase including CTX-M-15 resulting in broad spectrum resistance to β-lactam antibiotics [21]. Resistance to 3^rd^ generation cephalosporins in *Salmonella* has been attributed to the production of extended spectrum β-lactamases (ESBLs) [21]. The majority of invasive UGA isolates characterized in this study were susceptible to ceftriaxone, which therefore remains a viable treatment for invasive *Salmonella* infections. Further, only half of the isolates were susceptible to chloramphenicol, which indicates a reemergence of antibiotic susceptibility which has been observed by others [108].

Our study also identifies NTS isolates that display reduced cationic peptide sensitivity, which has been correlated with an increased sensitivity to the human innate immune system (i.e., human defense peptides) as an indicator of reduced virulence [92,109]. However, cationic peptide resistance has been shown to be required in *Salmonella* only during gastrointestinal infection, and not during bloodstream infections, indicating that although these UGA isolates can survive in blood during bacteremia, they would likely display reduced virulence when administered *via* the gastrointestinal route [110]. Management and mitigation of AMR through deliberate policy choices in antimicrobial availability and use may be a worthwhile pursuit [111]. Investigating the molecular basis of invasive NTS infections helps to elucidate the mechanism by which enhanced virulence and AMR evolve and support the development of accurate and effective countermeasures.

## Supporting information

S1 Table Illumina whole genome sequencing data. (PDF)

S2 Table Descriptions of isolates displayed in Fig 1. (PDF)

S3 Table Plasmids identified in each UGA isolate by sequencing. (PDF)

S1 Data Complete Biolog raw AUC data set. (XLSX)

S1 Fig Similarity between UGA14 plasmid pSLT-BT-UGA14 and reference *S*. Typhimurium plasmids pSBLT (ST313) and pSLT-BT (D23580). The AMR gene island is circled by dashed line, and the direct repeats in pSBLT are highlighted as yellow. There is a 19 kb inversion occurring in pSLT-BT but not in pSLT-BT-UGA14 (35838–54324 bps). There are two pSBLT genes, IS5075 and *repA*, that are not present in pSLT-BT-UGA14 at the 5’ and 3’ end of inversion boundaries, respectively. Although this hexameric replicative helicase RepA is missing in the UGA14 genome, IncFII family *repA* is present in pSLT-BT-UGA14, pSBLT and pSLT-BT. Notably, this 19 kb inversion region is overlapped with prophage regions from 52719–73452 and flanked by IS elements from 3 different backgrounds (pSBLT, pSLT-BT, and pSLT-BT-UGA14), indicating a potential recombination hotspot at this locus.
(PDF)

S2 Fig Comparison of AMR elements contained in *S*. Typhimurium plasmids pKST313-UGA14 and pSLT-BT-UGA14. (A) pKST313-UGA14 compared to plasmid pKST313 [21] and (B) pSLT-BT-UGA14 compared to plasmid pSLT-BT [28,73]. Homologous regions are indicated with a line in red (same direction) or blue (inverted). Homologous genes are in the same colored arrows, hypothetical proteins are in blue, and insertion sequence (IS) elements are in black (numbered as “1”, IS6 family; “2”, IS1; “3”, IS91; “4”, IS110; “5”, IS21; “6”, IS1380; “7”, IS3).
(PDF)

S3 Fig Comparison between UGA14 pSCP1-UGA14, pSA01AB09084001_4 and pPAB19-3. (PDF)

S4 Fig Comparison between UGA14 pSCP2-UGA14 and pRGRH0639. (PDF)

S5 Fig Comparison of plasmid pSLT-BT-UGA14 found in *S*. Typhimurium UGA10 with *S*. Enteritidis UGA18 p931-UGA14 with *S*. Anatum str. USDA-ARS-USMARC-1781 plasmid pSAN1-08-1092. (PDF)

S6 Fig Susceptibility of UGA isolates to (A) ampicillin/sulbactam (2/1) and (B) ciprofloxacin following growth in various media conditions. MHB: Mueller Hinton Broth; M9 minimal: M9 minimal media. Values plotted are mean ± standard deviation. MIC determinations represent the average of 2–4 independent experiments. Statistical significance was determined for UGA10 and UGA14 values compared to the reference strain ATCC 13311 with ***P < 0.001.
(PDF)
