# Supplementary material for: Comparative genomic and phenotypic characterization of invasive non-typhoidal Salmonella isolates from Siaya, Kenya
Source: PLoS Negl Trop Dis. 2021 Feb 1;15(2):e0008991. doi: 10.1371/journal.pntd.0008991 (PMC7877762; doi:10.1371/journal.pntd.0008991)
Supplement: S1 Table — (PDF) [file pntd.0008991.s001.pdf]

S1 Table. Illumina whole genome sequencing data.

Strain ID	Post-QC Illumina Draft Data (Mbp)	Estimated genome size (Mbp)	Average depth of coverage
UGA9	1603	4.94	324X
UGA10	1472	5.03	293X
UGA11	1363	4.94	276X
UGA12	1606	4.95	324X
UGA13	2436	4.95	492X
UGA14	2082	5,352,626 bp[a]	389.0X
UGA15	1760.	4.94	356X
UGA16	1520.	4.70	323X
UGA17	1971	4.95	398X
UGA18	1941	4.87	399X

[a] UGA14 is the only complete genome so actual genome size is reported and used for calculations of coverage
