# Supplementary material for: Comparative genomic and phenotypic characterization of invasive non-typhoidal Salmonella isolates from Siaya, Kenya
Source: PLoS Negl Trop Dis. 2021 Feb 1;15(2):e0008991. doi: 10.1371/journal.pntd.0008991 (PMC7877762; doi:10.1371/journal.pntd.0008991)
Supplement: S2 Table — (PDF) [file pntd.0008991.s002.pdf]

S2 Table. Descriptions of isolates displayed in Figure 1.

Isolate	Accession number	Year of isolation	Country of isolation	Host	Source	Phylogenetic lineage	Reference
356DRC	ERS004905	1991	DRC	Human	Blood	I	[24]
5580	ERS004896	2004	Kenya	Human	Blood	I	[24]
6325U	ERS009042	1998	Uganda	Human	Blood	I	[24]
A13198	ERS007457	2001	Malawi	Human	Blood	I	[24]
A13212	ERS007458	2001	Malawi	Human	Blood	I	[24]
M1111568	ERS009021	2001	Mozambique	Human	Feces	I	[24]
5582	ERS004957	2003	Kenya	Human	Blood	II	[24]
146U	ERS009045	2002	Uganda	Human	Blood	II	[24]
14DRC	ERS009029	1988	DRC	Human	Blood	II	[24]
A32773	ERS007466	2005	Malawi	Human	Blood	II	[24]
D23580	FN424405	2004	Malawi	Human	Blood	II	[24]
J3	ERS007479	2005	Mali	Human	Blood	II	[24]
M1605206	ERS007627	2002	Mozambique	Human	Blood	II	[24]
PO1140	ERS009026	2010	Nigeria	Human	Blood	II	[24]
Q258A	ERS033116	2002	Malawi	Human	Blood	II	[24]
UGA9	SRS7280280	2003	Kenya	Human	Blood	II	This study
UGA10	SRS7280281	2003	Kenya	Human	Blood	II	This study
UGA11	SRS7280283	2003	Kenya	Human	Blood	II	This study
UGA12	SRS7280284	2003	Kenya	Human	Blood	II	This study
UGA13	SRS7280285	2003	Kenya	Human	Blood	II	This study
UGA14	SRS7280286	2003	Kenya	Human	Blood	II	This study
UGA15	SRS7280287	2003	Kenya	Human	Blood	II	This study
UGA17	SRS7280289	2003	Kenya	Human	Blood	II	This study
UGA19	SRS7280282	2003	Kenya	Human	Blood	II	This study
249DRC	ERS004904	1991	DRC	Human	Blood	Other	[24]
5632	ERS004961	2003	Kenya	Human	Blood	Other	[24]
DT195	ERS007578	2009	UK	Human	Feces	Other	[24]
LT2	ERS007488	1946	UK	n.s.	n.s.	Other	[69, 70]
LT7	ERS007493	1946	USA	Lamb	n.s.	Other	[71]
LT8	ERS007494	1943	Denmark	Mouse	n.s.	Other	[71]
LT11	ERS007497	1938	Sweden	Rat	Feces	Other	[71]
Q303A	ERS033118	2002	Malawi	Human	Blood	Other	[24]
SARA4	ERS007502	1986	USA	Rabbit	n.s.	Other	[69, 70]
SARA5	ERS007503	n.s.	Mongolia	n.s.	n.s.	Other	[71]
SL1344	FQ312003	n.s.	UK	Bovine	GI	Other	[64]

UK: United Kingdom; n.s.: not specified; GI: gastrointestinal tract
