# Supplementary material for: Comparative genomic and phenotypic characterization of invasive non-typhoidal Salmonella isolates from Siaya, Kenya
Source: PLoS Negl Trop Dis. 2021 Feb 1;15(2):e0008991. doi: 10.1371/journal.pntd.0008991 (PMC7877762; doi:10.1371/journal.pntd.0008991)
Supplement: S3 Table — (PDF) [file pntd.0008991.s003.pdf]

S3 Table. Plasmids identified in each UGA isolate by sequencing.

Strain ID	pKST313-UGA14	pSLT-BT-UGA14	pSCP1-UGA14	pSCP2-UGA14	R64-type
UGA9		Contig 7, 12, 26		Contig 9	
UGA10		Contig 9, 10, 15, 32		Contig 8	Contig 51
UGA11		Contig 8, 12, 27	Contig 9	Contig 5	
UGA12		Contig 6, 8, 10, 16, 19, 27, 39	Contig 9	Contig 7	
UGA13		Contig 6, 8, 15, 18, 24	Contig 9	Contig 3	
UGA14	pKST313-UGA14	pSLT-BT-UGA14	pSCP1-UGA14	pSCP2-UGA14	
UGA15		Contig 10, 12, 14, 18, 24, 29			
UGA17		Contig 5, 13, 15, 34	Contig 10	Contig 7	
UGA19		Contig 8, 15, 17, 31, 49	Contig 6	Contig 5	
