# Supplementary figures and images for: Comparative genomic and phenotypic characterization of invasive non-typhoidal Salmonella isolates from Siaya, Kenya
Source: PLoS Negl Trop Dis. 2021 Feb 1;15(2):e0008991. doi: 10.1371/journal.pntd.0008991 (PMC7877762; doi:10.1371/journal.pntd.0008991)

S1 Fig

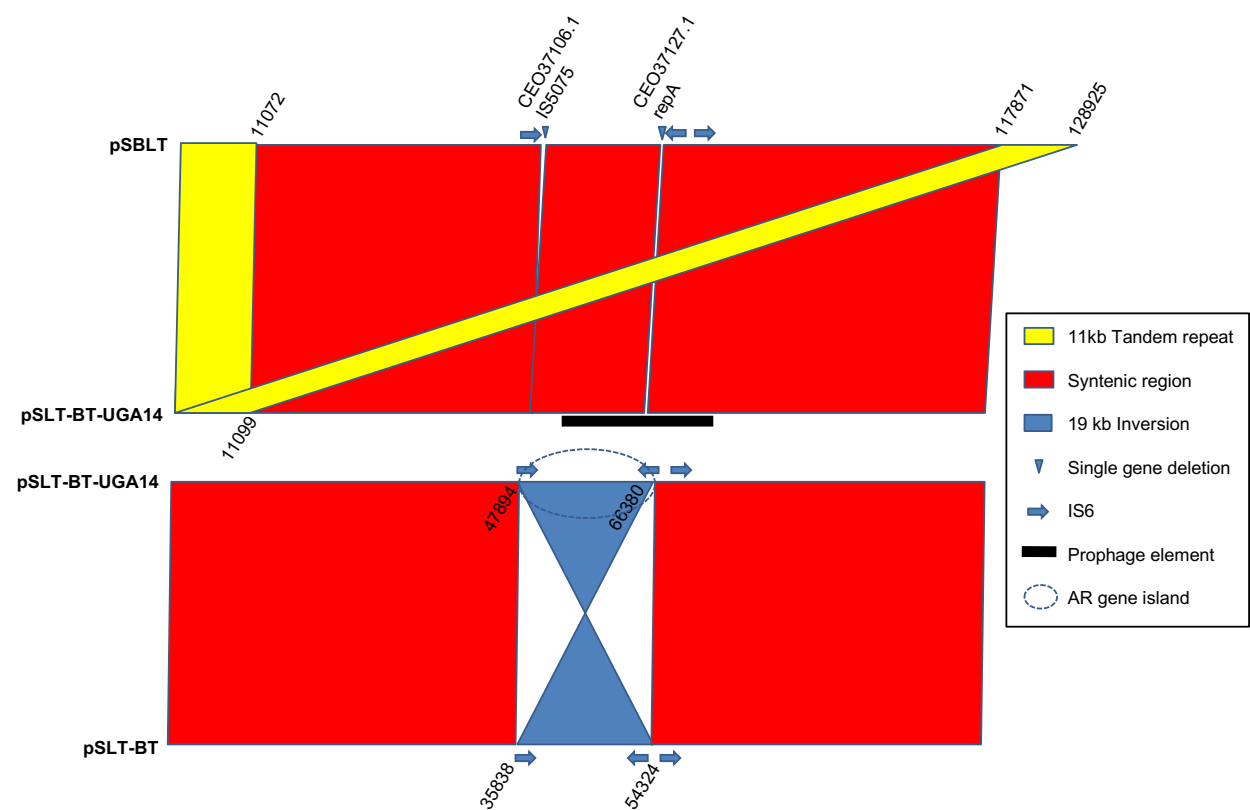

Supplement: S1 Fig — The AMR gene island is circled by dashed line, and the direct repeats in pSBLT are highlighted as yellow. There is a 19 kb inversion occurring in pSLT-BT but not in pSLT-BT-UGA14 (35838–54324 bps). There are two pSBLT genes, IS5075 and repA, that are not present in pSLT-BT-UGA14 at the 5’ and 3’ end of inversion boundaries, respectively. Although this hexameric replicative helicase RepA is missing in the UGA14 genome, IncFII family repA is present in pSLT-BT-UGA14, pSBLT and pSLT-BT. Notably, this 19 kb inversion region is overlapped with prophage regions from 52719–73452 and flanked by IS elements from 3 different backgrounds (pSBLT, pSLT-BT, and pSLT-BT-UGA14), indicating a potential recombination hotspot at this locus. (PDF) [file pntd.0008991.s005.pdf]

S2 Fig

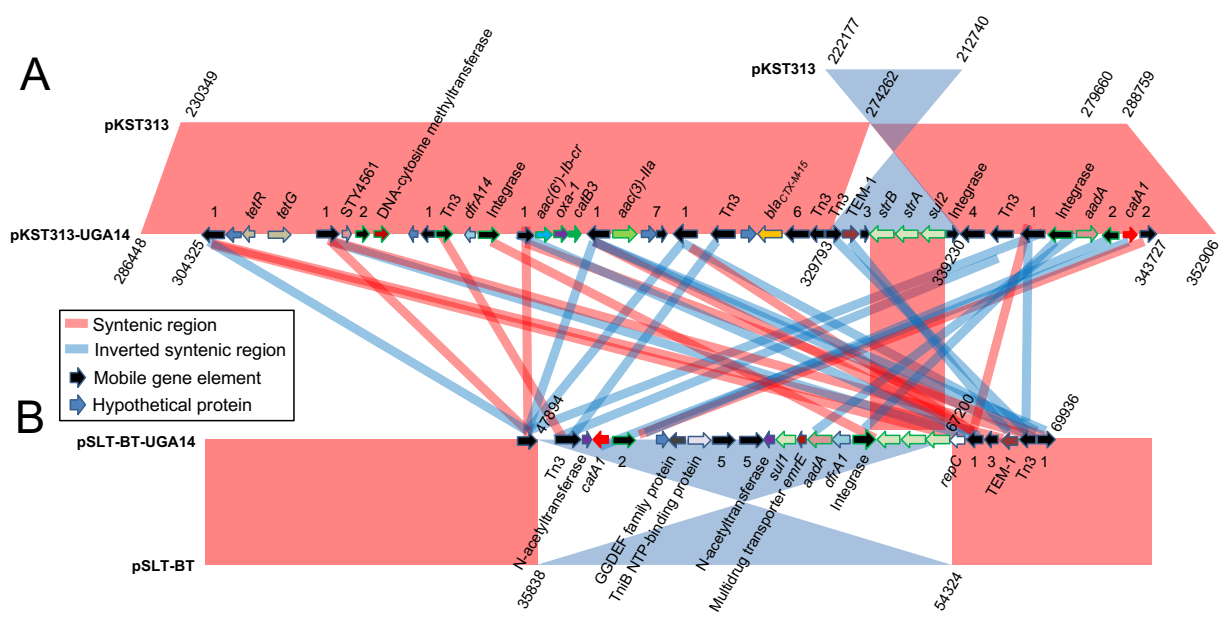

Supplement: S2 Fig — (A) pKST313-UGA14 compared to plasmid pKST313 [21] and (B) pSLT-BT-UGA14 compared to plasmid pSLT-BT [28,73]. Homologous regions are indicated with a line in red (same direction) or blue (inverted). Homologous genes are in the same colored arrows, hypothetical proteins are in blue, and insertion sequence (IS) elements are in black (numbered as “1”, IS6 family; “2”, IS1; “3”, IS91; “4”, IS110; “5”, IS21; “6”, IS1380; “7”, IS3). (PDF) [file pntd.0008991.s006.pdf]

S3 Fig

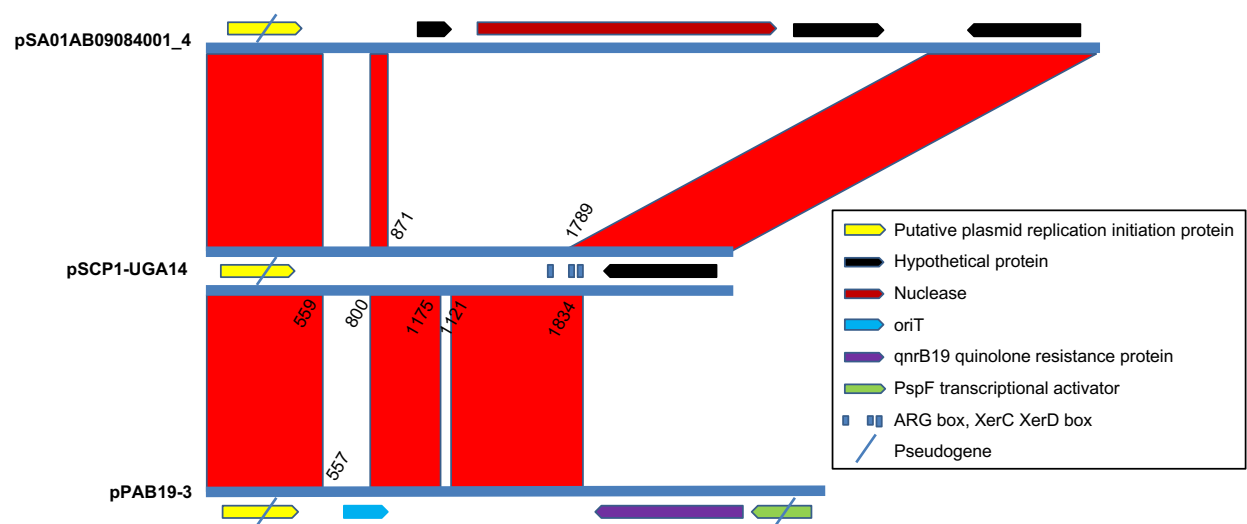

Supplement: S3 Fig — (PDF) [file pntd.0008991.s007.pdf]

S4 Fig

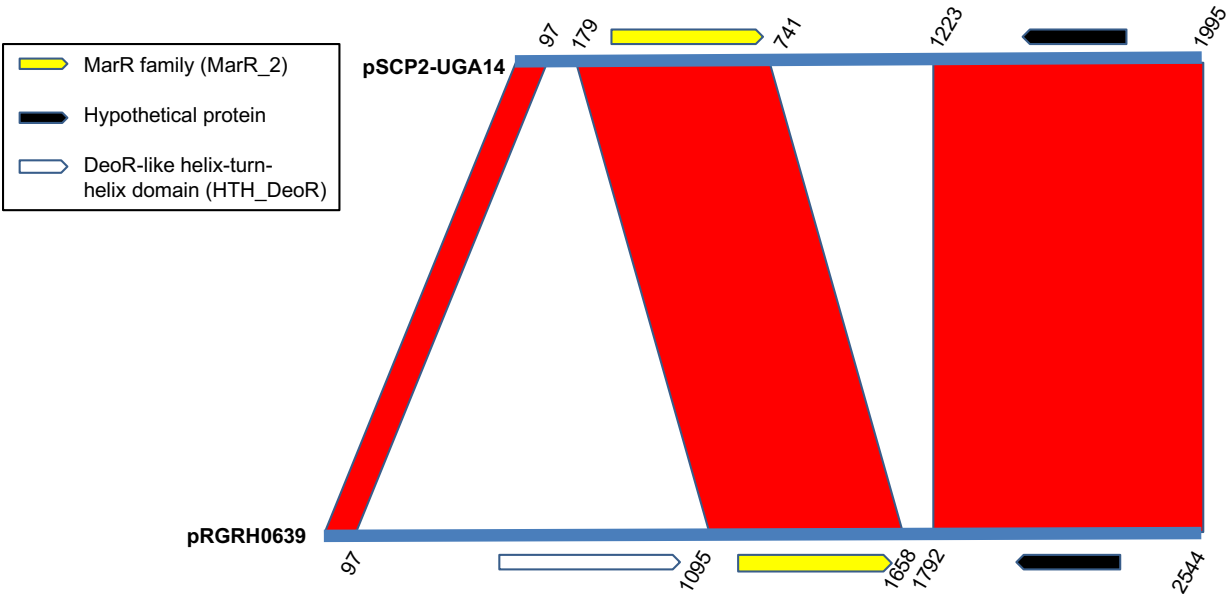

Supplement: S4 Fig — (PDF) [file pntd.0008991.s008.pdf]

S5 Fig

pSLT-BT-UGA14

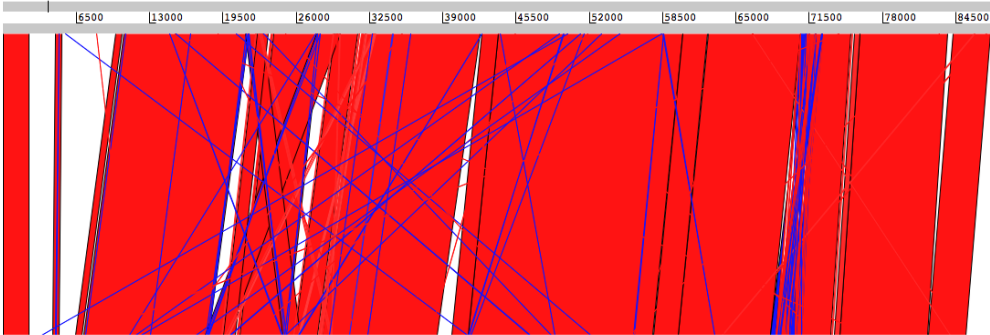

pSAN1-08-1092

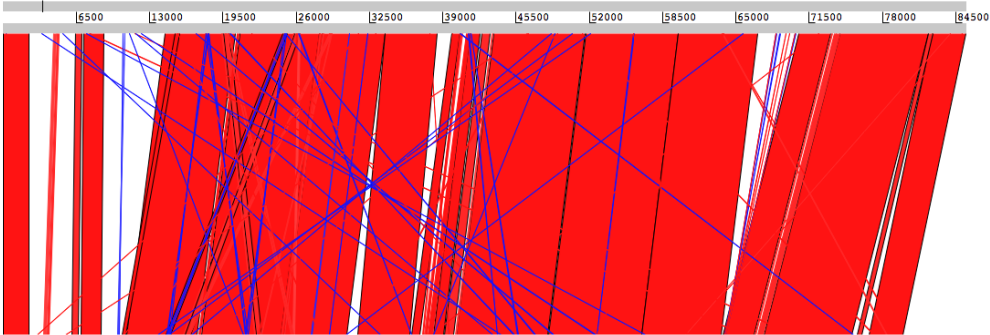

p931-UGA14

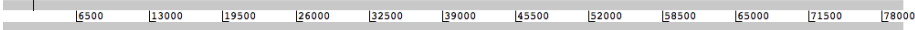

Supplement: S5 Fig — (PDF) [file pntd.0008991.s009.pdf]

S6 Fig

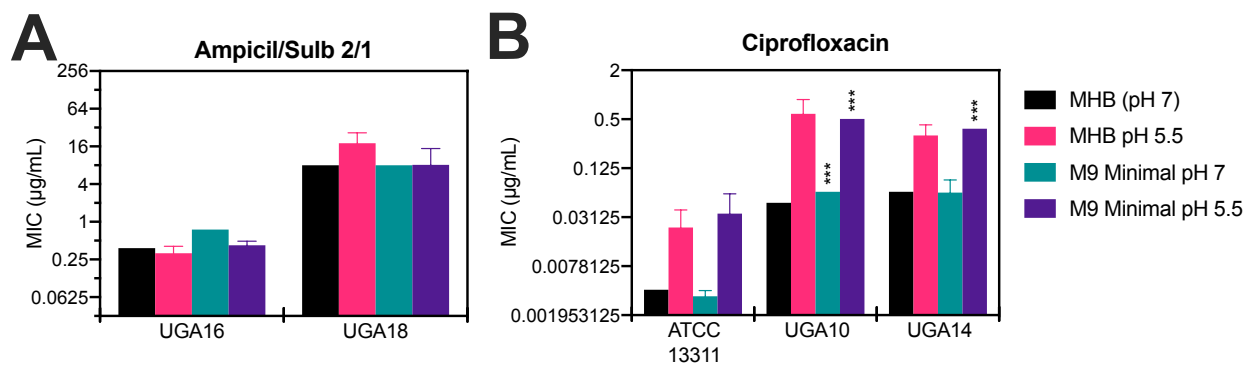

Supplement: S6 Fig — Susceptibility of UGA isolates to (A) ampicillin/sulbactam (2/1) and (B) ciprofloxacin following growth in various media conditions. MHB: Mueller Hinton Broth; M9 minimal: M9 minimal media. Values plotted are mean ± standard deviation. MIC determinations represent the average of 2–4 independent experiments. Statistical significance was determined for UGA10 and UGA14 values compared to the reference strain ATCC 13311 with ***P < 0.001. (PDF) [file pntd.0008991.s010.pdf]
